# A Smart Device System to Identify New Phenotypical Characteristics in Movement Disorders

**DOI:** 10.3389/fneur.2019.00048

**Published:** 2019-01-30

**Authors:** Julian Varghese, Stephan Niewöhner, Iñaki Soto-Rey, Stephanie Schipmann-Miletić, Nils Warneke, Tobias Warnecke, Martin Dugas

**Affiliations:** ^1^Institute of Medical Informatics, University of Münster, Münster, Germany; ^2^Department of Information Systems, University of Münster, Münster, Germany; ^3^Department of Neurosurgery, University Hospital Münster, Münster, Germany; ^4^Department of Neurology, University Hospital Münster, Münster, Germany

**Keywords:** Parkinson's Disease, Essential Tremor, smart wearables, artificial intelligence, neural networks

## Abstract

Parkinson's disease and Essential Tremor are two of the most common movement disorders and are still associated with high rates of misdiagnosis. Collected data by technology-based objective measures (TOMs) has the potential to provide new promising and highly accurate movement data for a better understanding of phenotypical characteristics and diagnostic support. A technology-based system called Smart Device System (SDS) is going to be implemented for multi-modal high-resolution acceleration measurement of patients with PD or ET within a clinical setting. The 2-year prospective observational study is conducted to identify new phenotypical biomarkers and train an Artificial Intelligence System. The SDS is going to be integrated and tested within a 20-min assessment including smartphone-based questionnaires, two smartwatches at both wrists and tablet-based Archimedean spirals drawing for deeper tremor-analyses. The electronic questionnaires will cover data on medication, family history and non-motor symptoms. In this paper, we describe the steps for this novel technology-utilizing examination, the principal steps for data analyses and the targeted performances of the system. Future work considers integration with Deep Brain Stimulation, dissemination into further sites and patient's home setting as well as integration with further data sources as neuroimaging and biobanks. Study Registration ID on ClinicalTrials.gov: NCT03638479.

## Introduction

Tremor-related diseases as Parkinson's Disease (PD) and Essential Tremor (ET) are two of the most common movement disorders (1). Disease classification is primarily based on clinical criteria and remains challenging (2, 3). Smart wearables with multi-sensor technology provide a source of objective movement monitoring allowing for greater precision in recording subtle changes unlike current clinical rating scales in hospital routine (4, 5). A technology-based system—called Smart Device System (SDS)—was implemented to monitor and visualize multi-modal high-resolution data. The project is going to be conducted in close collaboration with the local departments of Neurology and Neurosurgery and the Task Force on Technology by the International Movement Disorder Society. Though there is an increasing number of existent mobile apps from application stores or mature medical devices as the Parkinson's KinetiGraph™ system, there is a low number of large-scale deployments to capture and analyze the monitored data (6). More importantly, two key barriers—mentioned in a review by the International Movement Disorder Society—hamper integration into routine healthcare and identification of new phenotypical characteristics (7):

- First, there is poor integration of clinically relevant motor and non-motor phenomena. Especially, changes in tremor characteristics in relation to provoking circumstances (e.g., specific motor or cognitive actions) are currently not integrated into electronic tremor analyses. The SDS system utilizes different monitoring settings in one easy-to-follow examination at a health care facility: (A) Movement monitoring using two smartwatches at both patient wrists in a pre-defined neurological examination that provokes different tremor types. (B) Electronic questionnaires regarding medication/drug consumption, family history and non-motor symptoms on a smartphone. (C) Archimedean Spirals drawn by the patient on a tablet. Each of these three settings viewed individually already show high potential to classify or stratify tremor-related diseases (7–9). However, synchronization of these multimodal data (A–C) and advanced pattern recognition analyses such as Deep Learning would unlock a new integrative approach to boost classification performance and to find new phenotypical characteristics. In particular, Deep Learning methods have recently shown high classification performance in pattern recognition in other medical domains if a large amount of expert-tagged training data is available (10, 11).
- Second, there is a lack of open data repositories and data standards to share best practices of existing smart wearable solutions. This project uses the Medical-Data-Models Portal (MDM Portal) that has evolved to the largest European information infrastructure for medical data models (12). This portal will be used to harmonize and enrich developed data models toward established data standards and foster interoperability with other health information systems. Moreover, active collaboration with the Neurology Department and Task Force on Technology will align clinical and technical key requirements with current ongoing developments.

A 2-year prospective exploratory study is going to be conducted in which the SDS system will be applied within a 20-min data capture session for each included participant. The objective is to train a Deep Neural Network that is capable of predicting the disease entity (PD, ET, none of them) and to identify new phenotypical characteristics based on the fully integrated and pseudonymized study data.

## Materials and Equipment

The project is planned as a 2-year prospective exploratory study at the Movement Disorders outpatient clinic at the University Hospital Münster. Each patient diagnosed with PD (ICD 10-GM: G20.-) or ET (ICD-10-GM G25.0) and visiting the outpatient clinic is going to be a potential study participant. To be included, each patient must receive, fully understand and sign the informed consent form. All requirements of the updated EU data protection regulation from the start of May 2018 will be strictly adhered to. The study design is registered on Clinicaltrial.gov (Identifier: NCT03638479).

**Figure 1:**
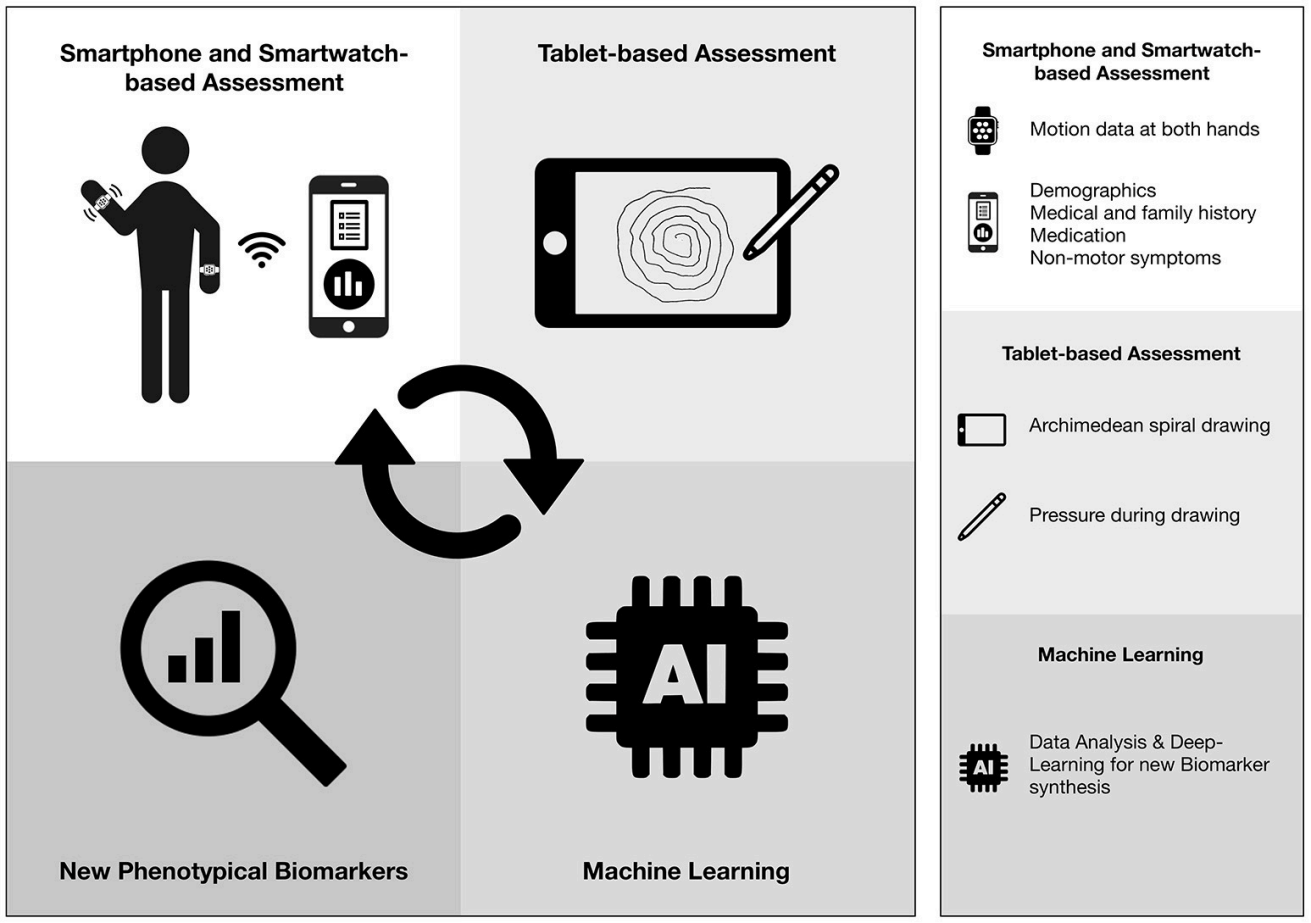
Overview of the Smart Device System.

Figure 1 provides an overview of the SDS system and its intended processing steps. Three Apple-based mobile devices will be customized for different monitoring settings. Smartwatch-based examination takes 10-min including calibration and putting them on both wrists. The total assessment time including questionnaires (8 min), drawing one spiral (1–2 min) is 20 min.

The examiner smartphone (iPhone at least Series 7) constitutes the first step of examination and provides short questionnaires about non-motor symptoms, family history and medication. Two smartwatches (Series 4.0) constitute the second step and enables high-resolution tremor capture from both wrists in a neurological examination (see Table 1). The captured data will be sent via Bluetooth to the smartphone. Since only one smartwatch can be paired with one smartphone at the same time, another smartphone is necessary to forward data from the second smartwatch to the examiner iPhone. The iPad Pro constitutes the final step of the assessment and will enable drawing of Archimedean spirals after protocol-based neurological examination. Captured data will be sent to the examiner smartphone, from which all captured data will be pseudonymized and securely transmitted in JSON format into a European General Data Protection Regulation-compliant research database. This connection to the research database is implemented within a REST-based client-server architecture using HTTPS as encryption. Two exemplary JSON files are attached as Supplementary Files that show structure of the transmitted raw data for two subject cases (one healthy subject, one with Parkinson's disease). The raw data from each JSON file will then be imported into a PostgreSQL-based research database. The overview of all data items is listed in the Table S1. All data processing, transmission, and storage processes were approved by the local data protection officer.

**Table 1:** Smartwatch-based steps of neurological examination.

**Step**	**Duration (s)**	**Description**
1a	20	Rest tremor. Participant is seated in resting position, standardized to Zhang et al. (9)
1b	20	Rest tremor and serial sevens. Participant starts from 100, subtracts 7, and stops after five answers.
2	10	Hold arms lifted.
3	10	Lift and extend arms according to Zhang et al. (9)
4	20	Hold 1 kg weight in every hand for 10 s. Start with the right hand. Then, have the participant's arm rested again as in 1a.
5	20	Finger pointing. Participant should point with his index fingers repetitively to examiner's lifted hand for 10 s. Start with participant's right index, then left.
6	20	Drink from glass. Have the participant grasp an empty glass with his right hand as if he/she would drink from it. Then repeat with his/her left hand.
7	10	Cross and extend both arms.
8	10	Bring both index fingers to each other, repeat until time expires.
9	20	Let participant's both index fingers tap his/her nose. Repetitively with the right (10 s), then with left index (10 s).
10	20	Entrainment. While holding the arms extended, have the participant stamp with his/her right foot according to the stamp frequency of the examiner. Then have him/her repeat with the left foot.

The software development for smartwatch-based and smartphone-based data capture is already finished. Tablet-based data capture will be included as soon as development and tests are completed. The first 3 months of the study consist of a setting up and testing phase, regardless of the tablet-based component. Only then, patients will be recruited and data capture commences and endures for 21 months. Based on a database report of the local EMR system, we expect 120 patients with ET and 954 patients with PD at our local site. A fixed number of follow up visits is not planned during this study. However, patients that will re-appear at the local site according to the usual treatment plan will be identified as follow-up patients to enable data analysis on disease progression.

## Stepwise Procedures

The following sections describe data capture settings in detail to provide a highly structured and reproducible data basis. Principal steps regarding data processing, analysis and model training are listed in the *Methods and Anticipated Results* section and will be subject to further refinements once the data is collected and evaluated.

### Recruitment

Each patients and his/her accompanying person (e.g., life partner), who visit the movement disorders outpatient clinic at the University Hospital in Münster and fulfill the aforementioned criteria will be asked to participate in the study. In most cases, each patient will be accompanied by a familiar person. These companions represent age-matched healthy controls. Each participant will be informed about the SDS system, the data to be captured, pseudonymized, transferred and stored for data analysis. Study inclusion starts once informed consent form has been signed.

### Data Capture and Neurological Examination

**Figure 2:**
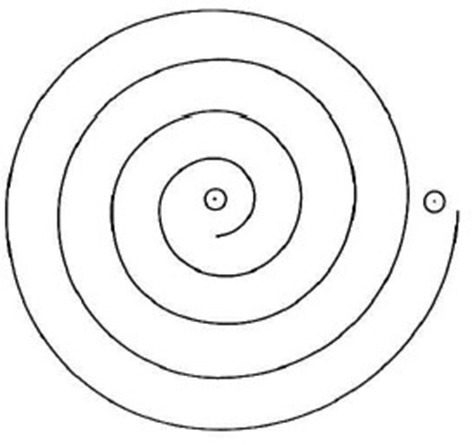
Archimedean spiral to be drawn with a pressure-sensing stylus on a 10.5 inch screen on an Apple iPad Touch, starting from the point in the middle.

Preceded by a literature review of tremor-related medical history, clinical phenomenology and the new tremor classification (8, 13–18) a series of workshops were conducted with neurologists specialized in Movement Disorders. As a result, a set of questionnaire items and a short technology-based examination were designed to capture data features, which were regarded to have highest predictive power for differential diagnoses of ET and PD. The questionnaire items will be captured first and are listed in Table S1. Then, neurological examination will start with one smartwatch at each participant's wrist, see Table 1. All steps in this examination are illustrated in a video (19) and the examiner initiates the next step or can repeat the current step. Thus, the acceleration data is always labeled with the corresponding examination step, which will provide essential context information for training the Deep-Learning model. The final assessment is to have the participant draw a spiral with a provided stylus and tablet (Figure 2), starting with the right hand and then with left hand. All components of the SDS-system (smartphone, smartwatches, and tablet) will only capture, pseudonymize, and submit data to a research database for Deep Learning. No components of the system will apply any analyses that intends to confirm or change any routine diagnostic or therapeutic procedures. Instead, all advanced data analyses will be conducted subsequently on the pseudonymized data at the research database server and none of analyses results will be sent back to the patient-level.

## Methods and Anticipated Results

**Figure 3:**
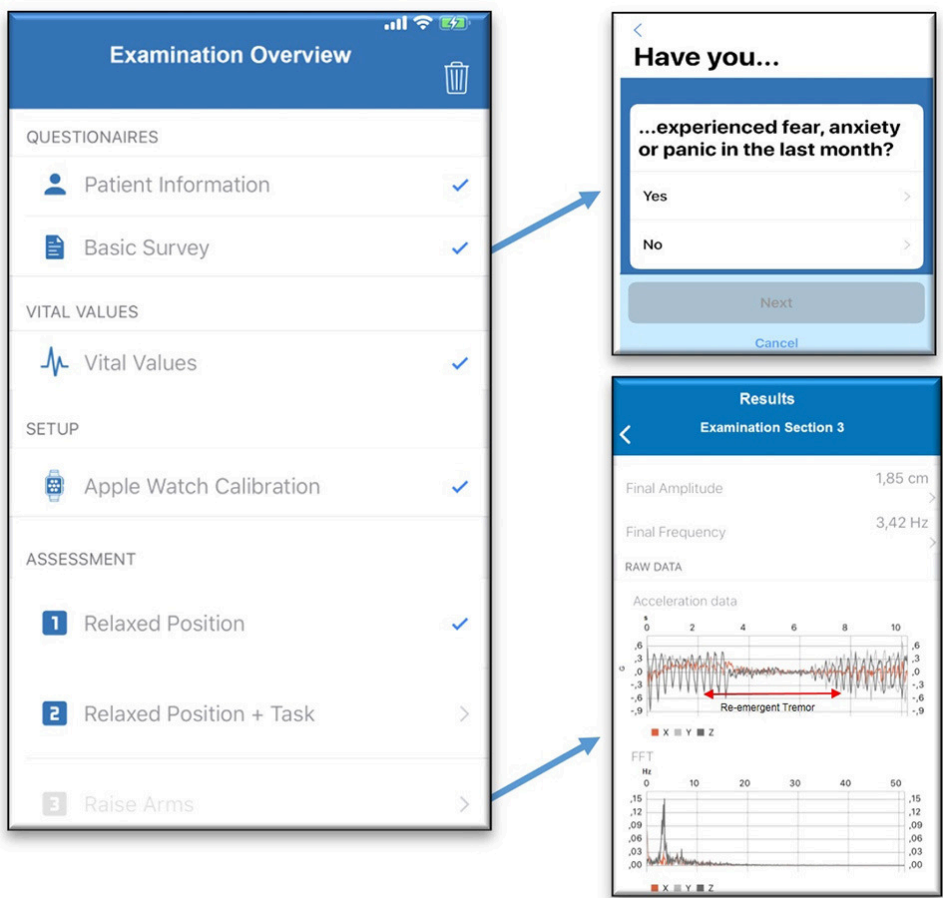
Overview screen **(left)**: the full examination takes approximately 20-min and starts with questionnaires and heart frequency measurement by the smartwatch. Before hand-tremor assessment starts, smart-watch calibration is required. Questionnaire screen **(upper right)**: showing one item of the PD-NMS instrument. Initial signal processing on the smartphone **(bottom right)**: visualization of average amplitude and frequency, raw acceleration data during examination section 3 and frequency spectrum. The acceleration data illustrates a simulated case of re-emergent tremor on the time axis. FFT, Fast Fourier Transform. Data capture and analyses of tablet-based spiral drawing is not implemented yet.

Acceleration data by the smartwatches will be analyzed with signal processing methods (including Fast Fourier Transform and band-pass filters) to infer average tremor amplitude and frequency in each examination section. Figure 3 illustrates a number of readily implemented graphical user interfaces and functioning initial results analyses.

Data captured by spiral drawing will be processed for angular feature detection, direction inversion and pattern deviation from ideal spiral according to Zham et al. (8). The sum of all captured and processed data will train a neural network model to predict the diagnosis label (PD, ET, none of them) and identify data patterns that could represent new biomarkers. The model will be trained applying Google Tensor Flow-based Network architectures using Convolutional neural networks (CNN) as they have shown highly promising pattern recognition results in medical images (11). In addition, long short-term memories (LSTM) networks will enable time-series analysis to account for temporal dynamics within the protocol-based examination and tablet-based spiral drawing. Both core methods (CNN + LSTM) will continuously be evaluated separately and in combination (e.g., using LSTM on top of CNN-extracted features). Sensitivity, positive predictive value/precision and accuracy will be calculated based on existent diagnosis labels using nested cross-validation. Statistical significance of classification results will be calculated by permutation tests. Univariate and Multivariate Analyses will be performed based on standard statistical regression analyses (e.g., logistic regression). This is necessary, first, to continuously and critically compare performance of Deep Learning black boxes with classical statistical approaches and second, to get a deeper understanding of the inter-related input features and their predictive power to classification results.

A concrete formulation of hypotheses is limited since we did not find any similar systems that were evaluated in studies from which we can derive a reliable patient sample size calculation based on statistical power analyses. Therefore, following key performance indicators (KPI) are aimed at the end of the study period based on classification performances in other disease domains (11, 20):

- **KPI 1:** >80% of all recruited participant cases will provide complete data as described in data capture Table S1 in the pseudonymized research database.
- **KPI 2:** >90% accuracy, >90% sensitivity, >90% positive predictive value for classifying ET and PD by the tested model using nested cross validation.

All data items from smartwatch-based data and questionnaires that are relevant for data analysis are specified in the standardized Operational Data Model by the clinical data interchange standards consortium and will be available on the MDM-Portal.

### Limitations

A proper sample size calculation was not performed, since it cannot be inferred how many samples the model will require to approach the targeted key performance indicators as—to our knowledge—there was no similar model trained and tested within this domain. However, there are basic assumptions of machine learning principles, stating that the size of training samples should be a multiple of the number input variables and output classes of the model to perform reasonably well (21). The smart-watch-based examination is the most data-consuming procedure capturing acceleration (A) and rotation (R) in 3 axes in 10 different examination sections at 2 different hands. This results in approximately 2 (A+R)^*^ 3 ^*^ 10 ^*^ 2 = 120 input variables. Three output classes are modeled (PD, ET, control: none of them or healthy). Ideally, we require a multiple of 120 ^*^ 3 training samples. Based on the EMR report, we can expect at least 500 patients with PD or ET at our local site, if every second will provide consent. Additionally, most of the patients will attend the ambulance with their spouses or partners, who will also be asked to participate as controls. We are convinced that the required recruitment number is approachable within the study phase, since the data capture is non-obtrusive without any interventional character.

In the case of reaching the targeted key performance indicators, they should still be taken with caution. The predictive capability will never be 100% flawless. This is even more likely to be the case if the system is tested in different environments with different examiners. The study will acquire at least two different regular examiners to train robustness for inter-examiner differences. We believe that this work has established a straight forward and fully-documented examination framework, that can be reproduced in other environments. After study completion, the resulting model, implementation code and execution description will be published as open source and supplied with anonymized training data samples.

## Interpretation of Anticipated Results

### Results

Each of the three technology-driven data capture settings is expected to show high potential to classify tremor-related diseases. Synchronization of these multimodal data and integrative pattern recognition analyses are expected to provide deeper insights into tremor characteristics. All of the technical devices used within this study have recently evolved to affordable mass products. Coupled with highly customizable apps, some of these devices already received FDA approval as for instance the Kardiaband app by AliveCor, the first FDA-cleared smartwatch-based ECG reader (22). This demonstrates the qualified use of such smart wearables not only for fitness or wellness purposes but also for valid medical use. A further advantage of the SDS system—compared to commercial and proprietary systems as the KinetiGraph™ system—is that all of its devices can be programmed and adapted by any Apple-based App developer through well-established Software Development kits and therefore preventing vendor lock-in. Once our system has reached the required level of accuracy, sensitivity and precision, regulatory steps will be taken for medical device approval and rollout to further sites. As a consequence, the system will not replace but could decisively extend diagnostic processes, which are currently suffering from high misdiagnoses rates in this domain (2, 3).

### Future Work

Deep brain stimulation (DBS) is an effective surgical treatment option and shows significant benefit for tremor and quality of life in patients with PD, ET, and other tremor etiologies (23–26). Intraoperative neurophysiological and clinical testing is important for optimal targeting of the DBS leads. Currently, evaluation of effects and symptoms is performed by an experienced neurologist and might be influenced by individual perception. Methods that enable an objective evaluation of the neurological status might help improving positioning of the DBS leads aiming at more accurate positioning and better results. A major challenge is to obtain medical clearance for the aforementioned devices or to establish sensors which comply with high regulatory requirements for intraoperative usage. In addition, the devices might help detecting minor changes in tremor pre- and post-operatively during the course of the disease and facilitate adjustment of DBS systems.

Extending the hospital-based setting, continuous and longitudinal measurement of movement and patient questionnaire-based input will be captured to provide patient home-based data for detailed monitoring of individual disease courses. This feature will be implemented after this study, because new phenotypical key characteristics from the IMF funded phase will first need to be identified and then captured with highest priority in the home-based setting. Moreover, the timely relations between medication intake and tremor effects will elucidate therapeutic effects in a long-term setting.

Further characteristics from Neuroimaging data are going to be identified and integrated to the research database. To elaborate on a larger patient sample size and ongoing genetic testing approaches of PD and ET, the need of a multi-center study and biobanks for genome-wide association analyses will be discussed.

## ETHICS STATEMENT

The ethical board of the University of Münster and the physician's chamber of Westphalia-Lippe approved the study protocol (Reference number: 2018-328-f-S).

## Author Contributions

JV has written the study design, acquired the funding, supervised the implementation of the system and written the manuscript. SN has implemented the system. IS-R has made significant contributions to the study design, acquisition of funding the project. SS-M and NW provided significant input to the study design and neurosurgical applications. TW has provided significant input to the study design, system requirements and the neurologic examination. MD has supervised and guided the project. All authors have received, critically revised and approved the manuscript.

### Conflict of Interest Statement

The authors declare that the research was conducted in the absence of any commercial or financial relationships that could be construed as a potential conflict of interest.
