# Supplementary material for: A Smart Device System to Identify New Phenotypical Characteristics in Movement Disorders
Source: Front Neurol. 2019 Jan 30;10:48. doi: 10.3389/fneur.2019.00048 (PMC6363699; doi:10.3389/fneur.2019.00048)
Supplement: Supplementary file 1 [file Table_1.DOCX]

Supplementary Material

A Smart Device System to Identify New Phenotypical Characteristics in Movement Disorders

Julian Varghese^*^

*** Correspondence:** Corresponding Author: email@uni.edu

# Table S1. Collection of data items.Data item table

| - **Type of data** | - **Description and purpose** |
| --- | --- |
| - Patient identification number in the local hospital information system of the university hospital | - Unambiguous number pseudonym, generated by the local hospital information. Ensures that all recorded data is assigned to this pseudonym. No identifying data such as names or dates of birth are saved. |
| - Current tremor diagnosis | - Documented on the smartphone by research personnel. Possible values: M. Parkinson as ICD-10-GM code, essential tremor as ICD-10-GM code, other tremor diseases as ICD-10-GM code or “no tremor disease”. Required to train a model for predicting the tremor diagnosis via deep learning. |
| - Age in years | - Documented in 10-year intervals on the smartphone by research personnel: 18-20, 20-29, 30-39, 40-49, 50-59, 60-69, 70-79, 80-89, 90-99, > 99. This enables age-dependent analyses. |
| - Sex | - Documented on the smartphone by research personnel. Biological sex, to enable sex-specific analyses. |
| - Body height and body weight | - Documented on the smartphone by research personnel. For calculating the BMI, which will be incorporated in the scientific evaluation. |
| - Rotation and acceleration | - Measured on both wrists by smart watch for motion analysis. |
| - Observations by study nurse in each examination section | - Documented on the smartphone: Arising Tremor? Re-emergent Tremor? Tremor with increasing amplitude? Further peculiarities regarding both hands and feet? - Head-Tremor? Jaw/Chin-Tremor? |
| - Pulse | - Measured on both wrists by smart watches as an influential variable for motion analyses. |
| - Body temperature | In-ear measurement by an existing medical device. |
| - Spiral drawing | - Measured pressure and writing of the spiral drawing on tablet (iPad Pro) recorded. Measurement enables pressure and motion analysis of both hands. |
| - Medication | - Documented on the smartphone by research personnel to consider the effects of medication on motion patterns. Recording of medication taken on the examination day, including active substance and dosage, and other medication, including active substance. |
| - Questionnaire: Information about deep brain stimulation, if carried out with study participant | - Documented on the smartphone by research personnel to consider operation-related influences on the motion analysis. Operation date, producer of the pace maker, stimulation kernel, stimulation parameters (amplitude, frequency, pulse width) recorded. |
| - Questionnaire: Family medical history | - Documented on the smartphone by research personnel to consider relations between tremors in the family and the own tremor disease. Incidences of M. Parkinson or essential tremor in family recorded? If so, is the first grade family affected? |
| - Questionnaire: PD NMS | - Documented on the smartphone by research personnel. Validated questionnaire composed of 30 yes/no items regarding non-motor symptoms. |
